# Mild hyponatremia, hypernatremia and incident cardiovascular disease and mortality in older men: A population-based cohort study

**DOI:** 10.1016/j.numecd.2015.07.008

**Published:** 2016-01

**Authors:** S.G. Wannamethee, A.G. Shaper, L. Lennon, O. Papacosta, P. Whincup

**Affiliations:** a Department of Primary Care and Population Health, UCL, London, UK; b Department of Population Health Sciences and Education, St George's, University of London, UK

**Keywords:** Serum sodium, Cardiovascular disease, Stroke, Mortality

## Abstract

**Aim:**

To examine the association between serum sodium concentration and incident major cardiovascular disease (CVD) outcomes and total mortality in older men.

**Methods and Results:**

A prospective study of 3099 men aged 60–79 years without a history of cardiovascular disease followed up for an average 11 years during which there were 528 major CVD events (fatal coronary heart disease [CHD] and non-fatal MI, stroke and CVD death) and 873 total deaths. A U shaped relationship was seen between serum sodium concentration and major CVD events and mortality. Hyponatremia (<136 mEq/L) and low sodium within the normal range (136–138 mEq/L) showed significantly increased risk of major CVD events and total mortality compared to men within the upper normal range (139–143 mEq/L) after adjustment for a wide range of confounders and traditional risk factors [adjusted HRs 1.55 (1.13,2.12) and 1.40 (1.14,1.72) for major CVD events respectively and 1.30 (1.02,1.66) and 1.30 (1.11,1.53) respectively for total mortality]. Hyponatremia was associated with inflammation, NT-proBNP, low muscle mass and alkaline phosphatase; these factors contributed to the increased total mortality associated with hyponatremia but did not explain the increased risk of CVD events associated with hyponatremia or low normal sodium concentration. Hypernatremia (≥145 mEq/L) was associated with significantly increased risk of CVD events and mortality due to CVD causes.

**Conclusion:**

Mild hyponatremia even within the normal sodium range and hypernatremia are both associated with increased total mortality and major CVD events in older men without CVD which is not explained by known adverse CV risk factors.

## Highlights

• Low serum sodium even within the normal range and hypernatremia are associated with increased risk of stroke and CVD.
• The association between low normal serum sodium and increased stroke and CVD risk is not explained by known CV risk factors.
• The presence of mild hyponatremia is not benign.
• By contrast no association is seen between serum potassium and risk of CVD.

## Introduction

Hyponatremia, usually defined as serum sodium concentration <136 mEq/L, is one of the most common electrolyte abnormalities observed in hospitalised patients [1] and in patients with chronic kidney disease (CKD), coronary heart disease (CHD) and heart failure (HF) [2], [3]. Several clinical and epidemiological studies have shown hyponatremia to be associated with increased total mortality in these patients [4], [5], [6]. In recent years, attention has turned to the possibility that mild hyponatremia, may be associated with adverse outcomes in the general population [7]. Studies on hyponatremia and mortality in community based populations are limited, but in the three population studies that have examined the relationship between hyponatremia and mortality in community based subjects, there is evidence that hyponatremia is associated with increased mortality [8], [9], [10] and that even a level of sodium concentration in the lower normal range (serum sodium 135–137 mEq/L), a level usually considered benign, is associated with increased mortality [9]. There is a paucity of data on electrolyte disturbance and risk of incident cardiovascular disease in the general population, but two studies have reported that low serum sodium, even within the normal range, is associated with increased stroke risk [11], [12], and possibly also to myocardial infarction [9]. Electrolyte disorders are common in the elderly [13] but less is known about the relationship between electrolyte disturbances and incident major CVD events and mortality in the general older population without established CVD. We have examined the association between serum sodium as well as serum potassium and risk of CHD, stroke, CVD mortality and total mortality in a population of older men aged 60–79 years with no history of CVD (CHD, HF or stroke).

## Methods

The British Regional Heart Study is a prospective study of cardiovascular disease involving 7735 men aged 40–59 years drawn from one general practice in each of 24 British towns, who were screened between 1978 and 1980 [14]. The population studied was socio-economically representative of British men and consisted almost entirely of white Europeans (>99%). In 1998–2000, all surviving men, now aged 60–79 years, were invited for a 20th year follow-up examination, on which the analyses presented here are based. Ethical approval was obtained from all relevant local research ethics committees. All men completed a mailed questionnaire providing information on their lifestyle and medical history, had a physical examination and provided a fasting blood sample collected using the Sarstedt Monovette system. The samples were frozen and stored at −20 °C on the day of collection and transferred in batches for storage at −70 °C until analysis, carried out after no more than one freeze–thaw cycle. The men were asked whether a doctor had ever told them that they had angina or myocardial infarction (MI), HF or stroke and to bring their medication to the examination session. 4252 men (77% of survivors) attended for the 1998–2000 examination and blood serum samples were available from 4088 men; 4034 men had measurements of sodium. Of these men, 935 men with pre-existing doctor diagnosed CHD (angina or MI), stroke or heart failure were excluded, leaving 3099 men for analyses.

### Cardiovascular risk factor measurements at 1998–2000

Details of measurement and classification methods for smoking status, physical activity, body mass index, social class, alcohol intake, blood pressure, blood lipids, alkaline phosphatase and forced expiratory volume in 1 s (FEV_1_) in this cohort have been described [15], [16], [17]. Mid arm muscle circumference (MAMC) calculated as mid upper arm circumference (MUAC) – 0.3142*(triceps skinfold thickness) was considered an indicator of muscle mass [16], [18]. Prevalent diabetes included men with a diagnosis of diabetes or men with fasting blood glucose ≥7 mmol/l. Predicted glomerular filtration rate (eGFR), estimated from serum creatinine using the equation developed by Levey et al. [19] was used as a measure of renal function. C-reactive protein (CRP) was assayed by ultra sensitive nephelometry (Dade Behring, Milton Keynes, UK) [20]. N-terminal pro-brain natriuretic peptide (NT-proBNP) was determined using the Elecsys 2010 electrochemiluminescence method (Roche Diagnostics, Burgess Hill, UK) [21].

#### Serum sodium

Sodium was measured by an ion selective electrode. A membrane composed of crown ether with a neutral PVC carrier forms a selective membrane for sodium ions, creating an electrical potential as sodium ions traverse the membrane [22]. The electrical potential can be compared to a reference electrode to determine the sodium ion concentration. The between batch imprecision was <2%.

#### Follow-up

All men have been followed up from initial examination (1978–1980) for cardiovascular morbidity [23] and follow-up has been achieved for 99% of the cohort. In the present analyses, total mortality and morbidity events are based on follow-up from re-screening in 1998–2000 at mean age 60–79 years to June 2010, a mean follow-up period of 11 years (range 10–12). Information on death was collected through the established “tagging” procedures provided by the National Health Service registers. Fatal stroke episodes were those coded on the death certificate to International Classification of Diseases (ICD-9th Revision) 430–438. Non-fatal stroke events were those which produced a neurological deficit that was present for more than 24 h. Fatal CHD events were defined as death with CHD (ICD 9th revision, codes 410–414) as the underlying code. A non-fatal MI was diagnosed according to World Health Organisation criteria [24]. Cardiovascular deaths included all those with ICD-9 codes 390–459. Evidence of non-fatal MI and HF was obtained by ad hoc reports from general practitioners supplemented by biennial reviews of the patients' practice records (including hospital and clinic correspondence) through to the end of the study period. Outcomes assessed in the current analyses were major CHD (defined as fatal or non-fatal MI) major stroke events (fatal or non-fatal), CVD death and all major CVD events (major CHD events, stroke events or CVD death).

#### Statistical methods

**Figure 1:**
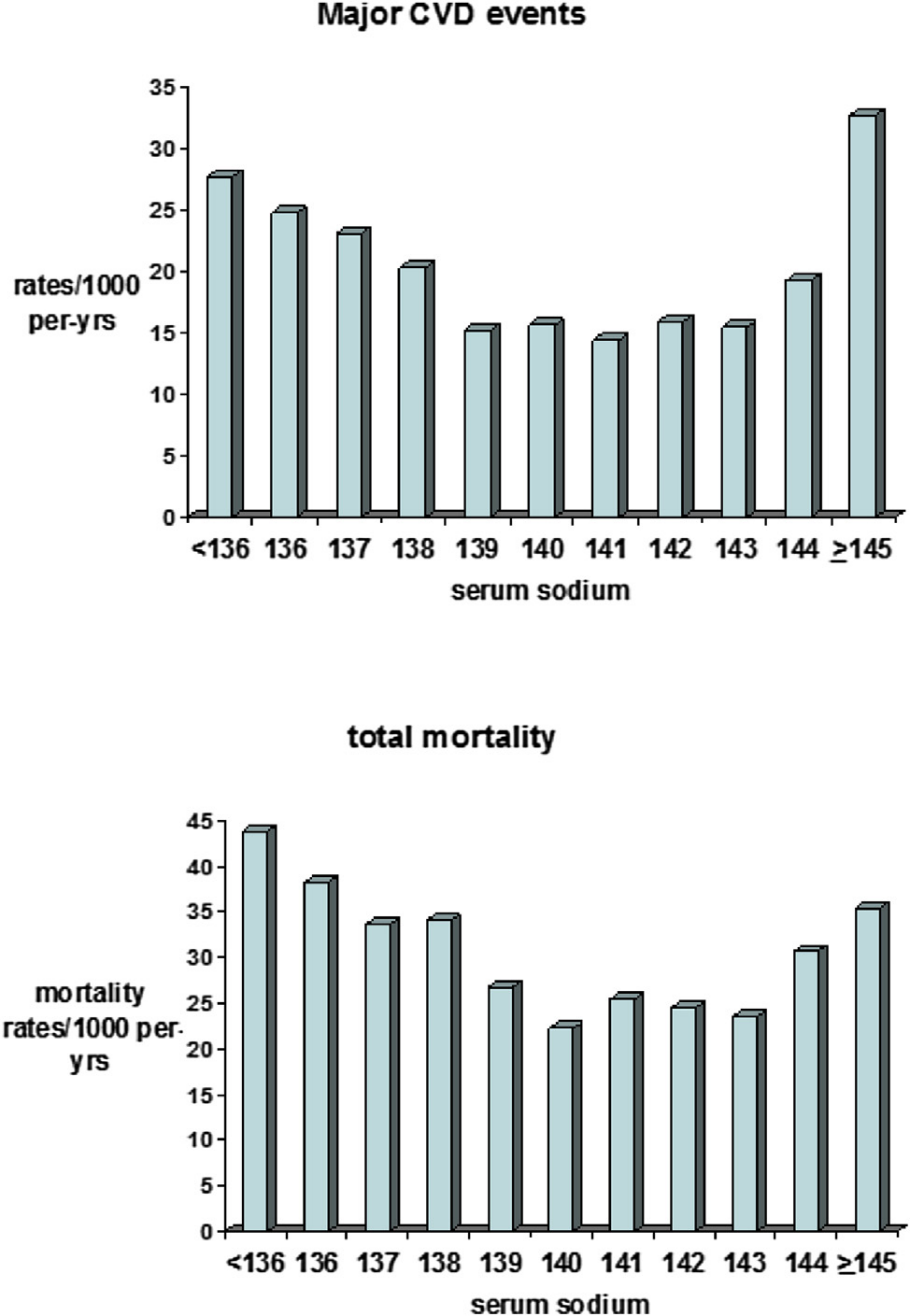
Serum sodium concentrations and incident cardiovascular rates/1000 person-years and total mortality rates/1000 person years. Number of men: Sodium levels <136 (n = 207), 136 (n = 134), 137 (n = 234), 138 (n = 330), 139 (n = 463), 140 (n = 494), 141 (n = 491), 142 (n = 337), 143 (n = 244), 144 (n = 109), ≥145 (n = 56).

Cox's proportional hazards model was used to assess the multivariate-adjusted hazards ratio (relative risk) by levels of serum sodium. Tests for quadratic trends in Fig. 1 were assessed by assigning quantitative values (1–11) for the 11 groups and fitting sodium as a continuous variable rather than as categorical variables and including a quadratic term. In multivariate analyses, smoking (never, long term ex-smokers (>15 years), recent ex-smokers (<15 years) and current smokers), social class (manual vs non manual), physical activity (4 groups), alcohol intake (5 groups), diabetes (yes/no), BMI (<25, 25–27.5, 27.5–29.9 and 30+ kg/m [2]), eGFR (<60, 60–69, ≥70 ml/min per 1.73 m^2^) and muscle mass (quartiles) were fitted as categorical variables; FEV1, HDL-C, CRP, systolic blood pressure, alkaline phosphatase, and NT-proBNP were fitted as continuous variables.

## Results

The mean serum sodium level in the 3099 men without CHD, stroke or HF was 139.7 mEq/L, SD (2.67) (range 125–150 mEq/L). Hyponatremia (<136 mEqu/L) was present in 6.7% of the men and hypernatremia (≥145 mEq/L) in 1.8% of men; only 15 men (0.5%) had levels above 145mEq/L. During the mean follow-up time of eleven years there were 269 CHD events, 209 stroke events and 293 CVD deaths, a total of 528 major incident CVD events (stroke, CHD, CVD deaths) in these men. Figure 1 shows the rates/1000 person years for overall CVD events and total mortality by levels of serum sodium concentration. A significant U-shaped relationship was seen with overall CVD events and total mortality with risk increasing below levels of 139 mEq/L and above 143 mEq/L (both p < 0.0001 for quadratic trend). On the basis of these findings we grouped the men into five categories: <136 (hyponatremia), 136–138 (low normal), 139–143, 144 and ≥ 145 (hypernatremia) mEq/L.

### Baseline characteristics by sodium

Table 1 shows the baseline characteristics by the five sodium groups. Hyponatremia was associated with older age and increased prevalence of smoking, heavier alcohol intake, diabetes and diuretic use. BMI and muscle mass tended to increase with increasing sodium levels. Hyponatremia was associated with the highest levels of CRP, NT-proBNP (marker of neurohormonal activation), GGT, alkaline phosphatase and eGFR and the lowest levels of FEV_1_. Overall, men with hypernatremia showed similar characteristics to those with normal sodium levels.

### Sodium and CVD risk

Men with sodium levels below 139 mEq/L and men with hypernatremia showed significantly higher risk of major CVD events than men with sodium levels between 139 and 143 mEq/L, even after adjustment for potential confounders, diabetes, blood pressure, blood lipids, lung function and eGFR (Table 2; model 1) and muscle mass (model 2). Further adjustment for CRP, alkaline phosphatase and NT-proBNP (model 3) made minor differences to the findings. When examined separately for CHD and stroke the increased risk associated with low sodium was seen for both CHD and stroke events and CVD death although hyponatremia related more to stroke events than CHD events. Hypernatremia related particularly strongly to stroke and CVD deaths. These associations persisted even after exclusion of men with renal dysfunction (eGFR <60), diuretic use and current smokers (N = 569) (Table 2).

### Sodium and total mortality

Hyponatremia and low sodium levels were associated with significantly increased mortality (Table 3). The increased risk of total mortality in men with hyponatremia was to some extent associated with muscle mass, CRP, alkaline phosphatase and NT-proBNP (Table 1). Men with hypernatremia showed increased risk in mortality compared to men with normal sodium levels, although the difference was not significant. The increased risk of mortality seen in those with low normal serum sodium was attenuated but remained significant even after exclusion of men with renal dysfunction (eGFR <60), diuretic use and current smokers (Table 3).

### Potassium and CVD and total mortality

**Figure 2:**
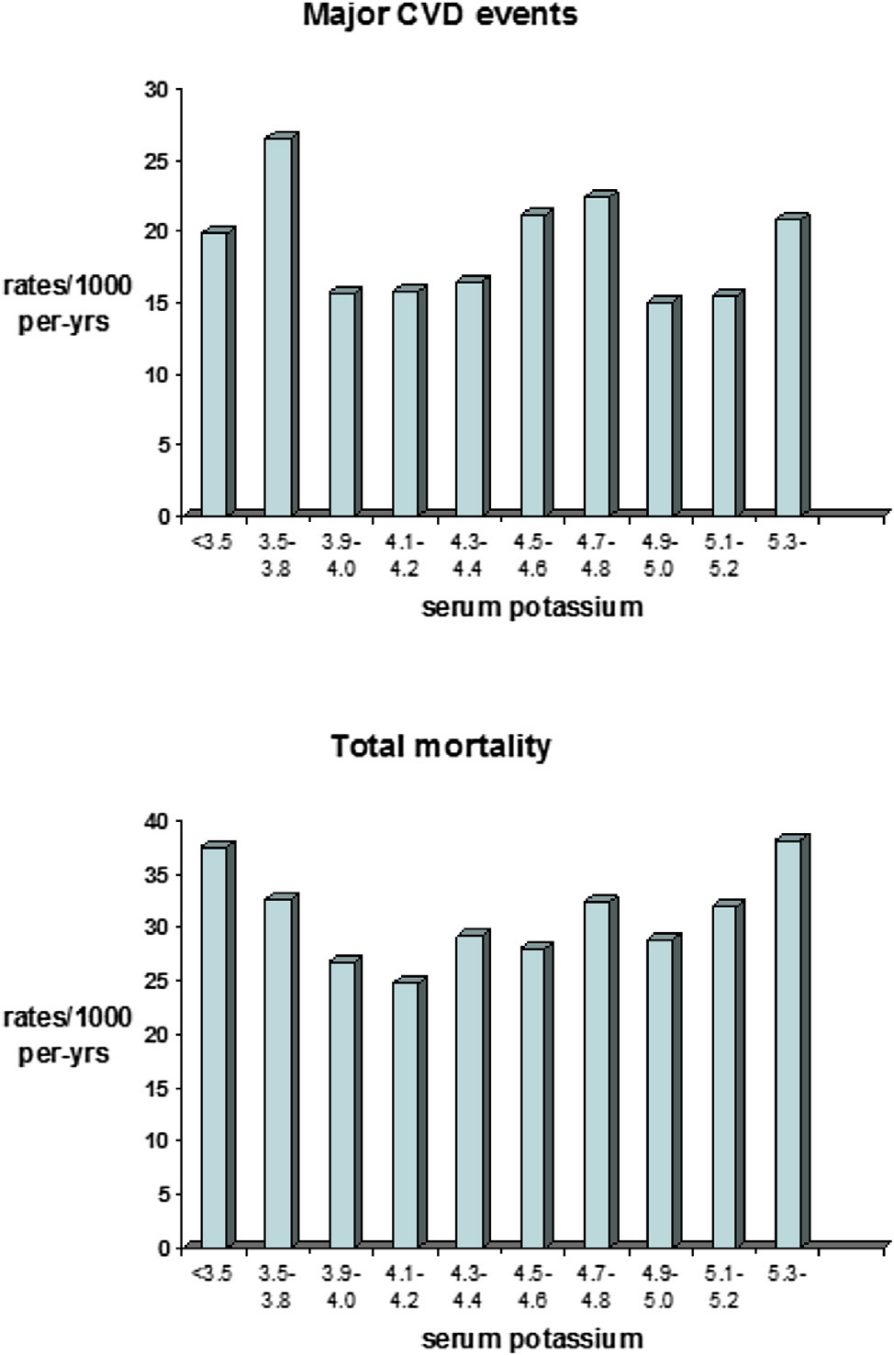
Serum potassium concentrations and incident cardiovascular rates/1000 person-years and total mortality rates/1000 person years. Number of men <3.5 (n = 12), 3.5–3.8 (n = 118), 3.9–4.0 (n = 341), 4.1–4.2 (n = 535), 4.3–4.4 (n = 664), 4.5–4.6 (n = 652), 4.7–4.8 (n = 209), 4.9–5.0 (n = 444), 5.1–5.2 (n = 68), ≥5.3 (n = 39).

Only 12 men were classified as having hypokalaemia (<3.5 mEq/L) and 107 men were classified as having hyperkalaemia (>5.0 mEq/L). In contrast to serum sodium, no consistent association was seen between potassium and overall CVD events (Fig. 2). A shallow but non-significant U shaped association was seen between potassium and total mortality with mortality highest in the very small groups of men with hypokalaemia and men with levels >5.3 mEq/L (n = 27) (Fig. 2).

## Discussion

In this study of older men without established CVD or HF, there was a U-shaped association between serum sodium and CVD events and mortality, with the lowest levels of risk in the 139–143 mEq/L group. Hyponatremia (<136 mEq/L) was present in about 6.7% of the population, a similar proportion to that observed in other healthy older populations [12]. Although hyponatremia is usually defined as levels <136 mEq/L [1], a more conservative definition of hyponatremia (sodium levels <138 mEq/L) was suggested by Kumar and Berl [25]. The significantly increased risk of CVD and mortality seen at circulating sodium levels between 136 and 138 mEq/L in the present study supports this more conservative definition and suggests that in older adults even levels between 136 and 138 mEq/L levels usually considered to be within the normal range (present in nearly a quarter of the men), may be a marker of mortality and CVD risk. Hypernatremia in this population was uncommon but was associated with significantly higher risk of both CVD events and increased CVD mortality. By contrast, serum potassium showed no significant association with total mortality or CVD events, which is consistent with a previous report [10]. Our study confirms other population studies that have shown mild hyponatremia to be associated with increased mortality [9] and extends the findings to cardiovascular events. We were able to examine a wide range of potential confounders and mediators in assessing the sodium CVD/mortality relationship including muscle mass, alkaline phosphatase (a biochemical marker of bone turnover) [17] and NT-proBNP (marker of neurohormonal activation).

### Low circulating sodium concentration and total mortality

In the majority of studies, predefined cut-off points have been used and comparisons are made between hyponatremic subjects and normonatremic subjects. We have shown that mortality decreases with increasing levels up to levels of 139 and levels out thereafter, rising again at levels above 144 (hypernatremia) which is similar to other studies conducted in hospital and CKD patients [6]. Hyponatraemia has several mechanisms, which could contribute to the relationship observed. Clinical studies have shown that neurohormonal activity (NT-proBNP), inflammation, diabetes and muscle mass are determinants of hyponatremia [3]. Recent studies suggest that hyponatremia could also affect other organs including lung and bone abnormalities [26], [27]. We have observed that hyponatremia is associated with several adverse risk factors including smoking, low muscle mass, low lung function, inflammation, increased levels of NT-proBNP, diabetes and increased levels of alkaline phosphatase, factors shown be associated with increased CVD and or mortality in this study [16], [17], [20], [20], [21]. These factors contributed to the increased total mortality risk in those with hyponatremia. However, they did not explain the increased total mortality risk seen at low sodium levels within the normal range.

### Low circulating sodium concentration and CVD risk

Few studies have examined the association between serum sodium and CVD. A previous report has suggested hyponatremia and low sodium (135–137 mEq/L), referred to as subtle hyponatremia, to be associated with increased risk of MI but the study was based on very small numbers with sodium levels (<138 mEq/L) (n = 76) [9]. Our study confirms a significant association between low sodium (but not hyponatremia) and major CHD (non-fatal MI and CHD deaths) which was not explained by known risk factors for CHD. The lack of association with CHD events with hyponatremia may be due to competing stroke events as these men showed exceptionally high risk of stroke. Our earlier report, conducted in middle-aged when the men were aged 40–59 years showed an association between low sodium and hypernatremia and increased risk of stroke [11]. This finding was confirmed in this population of men now aged 60–79 years and is consistent with findings from another cohort of older men showing sodium levels <140 mEq/L to increase stroke mortality risk [12]. The mechanisms underlying the relation between serum sodium and stroke are not clear, but it seems likely that different mechanisms are operating at the lower and higher ends of the serum sodium distribution. It is well recognized that acute changes in either direction cause cerebral disorders. Hyponatremia is known to affect the central nervous system and cause cerebral edema [28]. However, the increased risk of stroke was seen even within normal ranges, which was not explained by established risk factors for stroke.

The U shaped association between serum sodium and CVD is similar to the U-shaped association seen between dietary sodium intake as measured by urinary sodium excretion and CVD [29]. Although dietary sodium intake is shown to have a small effect on increasing circulating serum sodium levels [30], low circulating serum sodium under normal circumstances however, does not reflect low dietary sodium intake. The U-shaped association seen between serum sodium and CVD is thus unlikely to reflect dietary sodium intake. Speculatively, it is possible that the renin-angiotensin-aldosterone system (RAAS) might play an important role in the association between low sodium and CVD events. As a component of the RAAS system, aldosterone is an important mineralocortoid hormone involved in the regulation of fluid and electrolyte homeostatsis and has been associated with increased risk of CVD mortality [31]. Low sodium may have direct influence on CVD events by decreasing bone mineral density [26] which has been linked to increased risk of CVD [17]. Alternatively low normal serum sodium may represent a risk marker for poor health, rather than a risk factor for CVD or mortality. However, low normal serum sodium was related to CVD risk even in the absence of diuretic use, renal dysfunction and smoking.

### Hypernatremia

Hypernatremia is a common electrolyte disturbance in hospitalized patients and is well recognized to be associated with increased mortality [32]. Hypernatremia (≥145 mEq/L) was uncommon in this community dwelling older population without CVD or HF but was associated with significantly increased mortality largely due to CVD causes. The association seen more specifically for stroke which was not explained by diuretic use or renal dysfunction (as measured by the eGFR), common causes of hypernatremia, is consistent with the findings that hypernatremia is common in patients presenting with stroke at time of hospitalization [32].

### Strengths and limitations

Strengths and limitations of the study require consideration. This study is based on a cohort of older (60–79 year old) men who constitute a high risk group in whom electrolyte disturbances are common and in whom traditional risk factors are less predictive. Our results need confirmation both in similar older study populations and also in middle-aged populations and in particular women. The study population is socially representative of the UK, and follow-up rates in the British Regional Heart Study are exceptionally high. Ascertainment of CHD death and MI is based on standard methods and both CHD mortality and MI incidence rates correspond closely with national data. We were able to take into account a wide range of CV risk factors including markers of inflammation and cardiac markers. However, blood measurements were based on one measurement. Comparisons of sodium levels measured 20 years earlier in these men showed that 50% of those with low normal sodium levels (136–138 mEq/L) at baseline in 1978–1980 continued to have low normal or low serum sodium 20 years later while 50% had reverted to normal value. Overall tracking of sodium concentrations over the 20 year period was modest (r = 0.19) but highly statistically significant. However the fact that one sodium measure revealed an association with CVD risk and mortality suggests that the true association may be even stronger. Moreover, plasma aldosterone and renin, important determinants of electrolyte disorders were not measured in the study.

## Conclusion and implications

Hyponatremia and hypernatremia are both associated with increased risk of CVD incidence and mortality. Low sodium within the normal range is associated with significantly increased CVD events and total mortality in older men without major CVD or HF even in the absence of diuretic use and renal dysfunction. The data lends further evidence to the suggestion that the presence of mild hyponatremia is not benign. The findings may have important implications for the monitoring of sodium levels in clinical practice in older adults. The presence of mild hyponatremia in the absence of known causes such as renal dysfunction and diuretics may warrant further investigation in these men to assess CVD risk factors or possible underlying ill-health such as chronic inflammation. Further large studies are required to confirm and elucidate the nature of the association between low normal sodium and risk of incident CVD.

## Funding

The British Regional Heart Study is a British Heart Foundation (BHF) research group and receives support from BHF Programme grant RG/08/013/25942.

## Conflict of interest

None.

## Figures and Tables

**Table 1:** Baseline characteristics according to sodium levels (mEqu/L) in 3099 men with no diagnosed CHD, stroke or heart failure.

Sodium (mEq/L)						
	<136 (N = 207)	136-138 (N = 698)	139-143 (N = 2029)	144 (N = 109)	≥145 (N = 56)	P overall difference
Age (yrs)	69.8 (5.53)	68.6 (5.47)	68.0 (5.35)	68.6 (5.47)	68.2 (5.74)	<0.0001
% current smokers	18.0	14.9	12.2	10.2	7.3	0.04
% active	13.9	8.6	5.2	12.3	3.9	0.02
% manual	54.6	58.3	51.4	48.6	53.1	0.02
% moderate/heavy drinkers	22.7	19.5	19.3	9.2	12.5	0.03
% diabetes	15.9	16.2	7.8	15.9	8.9	<0.0001
% cancer	3.9	6.5	5.6	1.9	1.8	P = 0.19
% antihypertensive drugs	30.4	23.2	23.3	19.3	23.2	0.15
% diuretics	10.5	8.1	5.7	4.7	7.6	0.03
% statins	1.9	1.3	2.5	1.8	1.8	0.43
BMI (kg/m^2^)	25.8 (3.52)	26.8 (3.76)	26.8 (3.46)	27.4 (3.95)	27.1 (3.14)	0.001
Muscle mass (kg)	25.5 (2.51)	26.2 (2.29)	26.6 (2.33)	26.7 (2.13)	28.9 (2.29)	<0.0001
SBP (mmHg)	151.3 (23.8)	149.0 (22.6)	150.4 (24.1)	148.5 (24.3)	156.6 (25.1)	0.14
Cholesterol (mmol/l)	5.89 (1.14)	6.15 (1.06)	6.07 (1.05)	6.13 (1.03)	5.90 (1.26)	0.02
HDL-C (mmol/l)	1.38 (0.35)	1.35 (0.37)	1.33 (0.34)	1.32 (0.29)	1.27 (0.31)	0.11
Glucose*(mmol/l)	6.23 (5.26–6.25)	6.05 (5.31–6.29)	5.70 (5.24–6.02)	5.64 (5.20–5.89)	5.75 (5.17–5.79)	<0.0001
FEV_1_ (L)	2.40 (0.73)	2.60 (0.66)	2.67 (0.65)	2.54 (0.56)	2.68 (0.69)	<0.0001
CRP*(mg/L)	2.24 (0.88–5.55)	1.73 (0.78–3.59)	1.57 (0.78–3.09)	1.72 (0.89–3.75)	1.36 (0.70–2.85)	<0.0001
eGFR(ml/min per 1.73 m^2^)	76.1 (11.6)	73.4 (12.3)	72.7 (12.2)	69.1 (11.0)	73.5 (21.8)	<0.0001
NT-proBNP	109.9 (52–205)	81.5 (41–154)	80.6 (40–147)	90.0 (42–182)	93.7 (50–202)	0.004
ALP	86.5 (71–108)	79.8 (66–94)	80.6 (68–94)	85.6 (67–99)	83.1 (69–95)	0.006
GGT	33.1 (20–44)	28.8 (19–38)	27.7 (19–38)	27.1 (19–38)	27.7 (18–32)	0.0007
Blood urea	5.52 (1.52)	5.88 (1.73)	5.98 (1.51)	5.89 (1.26)	6.17 (1.42)	0.001

Mean and SD; * geometric mean and interquartile range.

**Table 2:** Incidence rates/1000 person years and adjusted hazards ratios (95%CI) for major cardiovascular events by serum sodium levels (mEqu/L) in 3099 men without CHD, stroke or heart failure.

Serum sodium (mEq/L)					
	<136 (N = 207)	136-138 (N = 698)	139-143 (N = 2029)	144 (N = 109)	≥145 (N = 56)
All CVD events (n = 528)					
Rate/1000 per-yrs	27.7 (49)	22.1 (140)	15.3 (303)	19.3 (20)	32.7 (16)
Age-adjusted	1.50 (1.11,2.07)	1.42 (1.16,1.73)	1.00	1.20 (0.76,1.88)	2.20 (1.33,3.63)
Model 1	1.55 (1.13,2.12)	1.40 (1.14,1.72)	1.00	1.24 (0.78,2.00)	2.24 (1.33,3.65)
Model 2	1.52 (1.10,2.09)	1.38 (1.12,1.69)	1.00	1.23 (0.77,2.00)	2.16 (1.30,3.50)
Model 3	1.48 (1.08,2.04)	1.43 (1.16,1.76)	1.00	1.20 (0.75,1.91)	2.07 (1.23,3.42)
(with exclusion^a^)	1.46 (1.01,2.10)	1.31 (1.02,1.67)	1.00	1.01 (0.56,0.82)	2.38 (1.31,4.32)
CHD events (n = 269)					
Rate/1000 per-yrs (n)	10.7 (20)	11.2 (73)	7.8 (158)	10.4 (11)	13.2 (7)
Age-adjusted	1.17 (0.73,1.86)	1.40 (1.06,1.85)	1.00	1.28 (0.70,2.36)	1.71 (0.80,3.64)
Model 1	1.19 (0.73,1.94)	1.39 (1.05,1.85)	1.00	1.36 (0.74,2.52)	1.71 (0.80,3.66)
Model 2	1.14 (0.70,1.86)	1.34 (1.01,1.79)	1.00	1.40 (0.76,2.59)	1.69 (0.79,3.62)
Model 3	1.18 (0.72,1.93)	1.45 (1.09,1.94)	1.00	1.37 (0.74,2.54)	1.63 (0.76,3.50)
(with exclusion^a^)	1.20 (0.69,2.10)	1.46 (1.04,2.04)	1.00	1.03 (0.45,2.35)	1.68 (0.67,4.21)
Stroke events (n = 209)					
Rate/1000 per-yrs	15.6 (28)	8.1 (52)	5.7 (116)	5.7 (6)	14.2 (7)
Age-adjusted	2.29 (1.51,3.48)	1.38 (1.00,1.92)	1.00	0.94 (0.42,2.15)	2.54 (1.19,5.46)
Model 1	2.30 (1.49,3.55)	1.43 (1.02,2.00)	1.00	0.86 (0.35,2.11)	2.60 (1.21,5.62)
Model 2	2.28 (1.48,3.53)	1.43 (1.02,2.00)	1.00	0.86 (0.35,2.11)	2.57 (1.19,5.54)
Model 3	2.12 (1.37,3.28)	1.42 (1.02,2.50)	1.00	0.83 (0.34,2.04)	2.49 (1.15,5.39)
(with exclusion^a^)	2.02 (1.23,3.32)	1.14 (0.76,1.71)	1.00	0.80 (0.29,2.18)	2.58 (1.04,6.45)
CVD deaths (n = 293)					
Rate/1000 per-yrs	15.3 (29)	12.8 (85)	7.6 (156)	13.0 (14)	16.7 (9)
Age-adjusted	1.55 (1.04,2.30)	1.62 (1.24,2.11)	1.00	1.63 (0.94,2.81)	2.25 (1.15,4.41)
Model 1	1.60 (1.05,2.43)	1.58 (1.20,2.08)	1.00	1.77 (1.00,3.14)	2.38 (1.21,4.69)
Model 2	1.53 (1.01,2.34)	1.53 (1.16,2.01)	1.00	1.81 (1.02,3.26)	2.32 (1.17,4.57)
Model 3	1.61 (1.05,2.46)	1.64 (1.24,2.16)	1.00	1.73 (0.98,3.08)	2.25 (1.14,4.47)
(with exclusion^a^)	1.68 (1.03,2.73)	1.58 (1.12,2.22)	1.00	1.19 (0.52,2.73)	2.57 (1.09,6.03)

Model 1 = adjusted for age, cigarette smoking, alcohol intake, physical activity, social class, BMI use of antihypertensive drugs, diabetes, lung function, systolic blood pressure and eGFR.

Model 2 adjusted for Model 1 + muscle mass.

Model 3 Adjusted for Model 1 + CRP + alkaline phosphatase + NT-proBNP.

a Exclude men with diuretic use, renal dysfunction (eGFR<60) and current smokers.

**Table 3:** Mortality rates/1000 person years (number of deaths) and adjusted hazards ratios (95%CI) for total mortality in men without CHD, stroke or heart failure.

Serum sodium (mEq/L)					
	<136 (N = 207)	136–138 (N = 698)	139–143 (N = 2029)	144 (N = 109)	≥145 (N = 56)
Total mortality (n = 873)					
Rate/1000 per-yrs (n)	43.9 (83)	34.8 (230)	24.6 (508)	30.7 (33)	35.4 (14)
Age-adjusted	1.47 (1.16,1.85)	1.39 (1.19,1.62)	1.00	1.19 (0.84,1.69)	1.48 (0.94,2.34)
Model 1	1.30 (1.02,1.66)	1.30 (1.11,1.53)	1.00	1.25 (0.87,1.78)	1.54 (0.97,2.44)
Model 2	1.27 (0.99,1.62)	1.27 (1.08,1.49)	1.00	1.27 (0.88,1.81)	1.52 (0.96,2.41)
Model 3	1.24 (0.97,1.59)	1.33 (1.13,1.56)	1.00	1.20 (0.83,1.73)	1.46 (0.92,2.32)
(With exclusion^a^)	1.23 (0.94,1.63)	1.22 (1.01,1.47)	1.00	1.20 (0.83,1.73)	1.53 (0.85,2.75)

Model 1 = adjusted for age, cigarette smoking, alcohol intake, physical activity, social class, BMI, use of antihypertensive drugs, diabetes, lung function, systolic blood pressure and eGFR.

Model 2 = Model 1 + muscle mass.

Model 3 = Model 1 + muscle mass + CRP + alkaline phosphatase + NT-proBNP.

a Exclude men with diuretic use, renal dysfunction (eGFR<60) and current smokers.
